# The Complete Mitochondrial Genome Sequence of *Bactericera cockerelli* and Comparison with Three Other Psylloidea Species

**DOI:** 10.1371/journal.pone.0155318

**Published:** 2016-05-26

**Authors:** Fengnian Wu, Yijing Cen, Christopher M. Wallis, John T. Trumble, Sean Prager, Ray Yokomi, Zheng Zheng, Xiaoling Deng, Jianchi Chen, Guangwen Liang

**Affiliations:** 1 Laboratory of Insect Ecology / Guangdong Province, Key Laboratory of Microbial Signals and Disease Control, College of Agriculture, South China Agricultural University, Guangzhou, Guangdong, China; 2 San Joaquin Valley Agricultural Sciences Center, United States Department of Agriculture -Agricultural Research Service, Parlier, California, United States of America; 3 Department of Entomology, University of California Riverside, Riverside, California, United States of America; University of Idaho, UNITED STATES

## Abstract

Potato psyllid (*Bactericera cockerelli*) is an important pest of potato, tomato and pepper. Not only could a toxin secreted by nymphs results in serious phytotoxemia in some host plants, but also over the past few years *B*. *cockerelli* was shown to transmit “*Candidatus* Liberibacter solanacearum”, the putative bacterial pathogen of potato zebra chip (ZC) disease, to potato and tomato. ZC has caused devastating losses to potato production in the western U.S., Mexico, and elsewhere. New knowledge of the genetic diversity of the *B*. *cockerelli* is needed to develop improved strategies to manage pest populations. Mitochondrial genome (mitogenome) sequencing provides important knowledge about insect evolution and diversity in and among populations. This report provides the first complete *B*. *cockerelli* mitogenome sequence as determined by next generation sequencing technology (Illumina MiSeq). The circular *B*. *cockerelli* mitogenome had a size of 15,220 bp with 13 protein-coding gene (PCGs), 2 ribosomal RNA genes (rRNAs), 22 transfer RNA genes (tRNAs), and a non-coding region of 975 bp. The overall gene order of the *B*. *cockerelli* mitogenome is identical to three other published Psylloidea mitogenomes: one species from the Triozidae, *Paratrioza sinica*; and two species from the Psyllidae, *Cacopsylla coccinea* and *Pachypsylla venusta*. This suggests all of these species share a common ancestral mitogenome. However, sequence analyses revealed differences between and among the insect families, in particular a unique region that can be folded into three stem-loop secondary structures present only within the *B*. *cockerelli* mitogenome. A phylogenetic tree based on the 13 PCGs matched an existing taxonomy scheme that was based on morphological characteristics. The available complete mitogenome sequence makes it accessible to all genes for future population diversity evaluation of *B*. *cockerelli*.

## Introduction

The potato, or potato-tomato, psyllid *Bactericera cockerelli* (Šulc) (Hemiptera: Triozidae) is an important pest of potato, tomato and pepper production. The insect can cause damage on plants when feeding via phytotoxemia, and, in addition, also can transmit “*Candidatus* Liberibacter solanacearum” (a.k.a. “*Ca*. L. psyllaurous”), an unculturable alpha-proteobacterium associated with potato zebra chip (ZC) disease [1, 2]. *B*. *cockerelli* was first described over a hundred years ago under the name of *Trioza cockerelli* [3]. Thus, for over a century substantial efforts have been made to study the taxonomy, damage mechanism, and management for this psyllid [4]. However, knowledge is limited on *B*. *cockerelli* evolution and population diversity.

*B*. *cockerelli* was thought to annually migrate from warm southern regions of North America (e.g. Mexico) to the western United States (e.g. North Texas, Colorado, the Dakotas, Kansas, Nebraska) via air currents, where it then colonizes and damages solanaceous crop plants [5–7]. However, the existence of distinct regional populations of *B*. *cockerelli* (biotypes) were discovered following a 2001 outbreak throughout western North America, based on variations revealed by inter simple sequence repeat (ISSR) markers and single nucleotide polymorphism (SNP) in the mitochondrial gene of cytochrome oxidase I (*cox1*) [8]. That study determined *B*. *cockerelli* populations clustered into two biotypes, one from western North America and one from the central United States of America (USA) and eastern Mexico. Existence of *B*. *cockerelli* biotypes suggests adaptation to local habitats are taking place, hence, there may be less dependence on long-range dispersal and a concomitant increase in the regional pest status associated with *B*. *cockerelli* populations.

Later, Swisher et al. identified three haplotypes of *B*. *cockerelli* within USA based on their SNP analyses of a 500-bp *cox1* gene sequence: a Central haplotype from eastern Mexico north to Texas, Kansas, Colorado, Nebraska, Wyoming, and North Dakota; a Western haplotype, from California and New Mexico north to Washington, Oregon, and Idaho; and a Northwestern haplotype, including the Northwestern states of Washington, Oregon, and Idaho [9]. More recently, an additional haplotype (Southwestern) was identified and is found in New Mexico and southern Colorado [10]. However, some haplotypes (Central and Western) were differentiated by only one SNP. The same SNP was also described by Liu *et al* [8] in their biotype study. To assure a comprehensive description and haplotype/biotype stability of the *B*. *cockerelli*, we feel that there is a need to evaluate more sequences from the *cox1* and other genes in the psyllid genome.

In the recent years, whole genome sequencing of mitochondria genomes (mitogenomes) has gained importance for comprehensive evolutionary and population studies of insects. This is due to the relatively small genome sizes, low levels of recombination, and variable evolution rates of genes in the mitogemones. With few exceptions, insect mitogenomes consist of 13 protein-coding genes (PCGs), two ribosomal RNA genes (rRNA), 22 transfer RNA genes (tRNA), and a large non-coding region (also called the control region, CR) on a single circular chromosome. Arrangement of genes in mitogenomes is usually stable, retaining the ancestral pattern of gene arrangement [11]. Previous mitogenome sequencing has been heavily based on sequencing DNAs amplified using conserved PCR primers by the Sanger method[12–16]. This method is both time consuming and laborious. However, presently Next-Generation Sequencing (NGS) technologies have been developed to generate a large amount of short DNA sequences (reads) from a single insect. Mitochondrial reads can then be found based on appropriate reference sequences and used to assemble complete mitogenome sequences [17, 18].

In the superfamily Psylloidea, three complete mitogenome sequences have been published and available for public use in GenBank database: *Paratrioza sinica* in the family of Triozidae [19], and *Cacopsylla coccinea* and *Pachypsylla venusta* in the family of Psyllidae [12, 16]. *P*. *sinica* is an important pest of wolfberry or Goji (*Lycium chinense*) in Northwest China, where feeding on tender shoots and buds of the host causes yellow leaves and declined growth [20]. *C*. *coccinae* is a pest of chocolate vine (*Akebia quinata*) in China, Japan and Korea [20]. *P*. *venusta* causes damage to hackberry (*Celtis occidentalis*) in the U.S. by forming woody galls on leaf petioles [21]. Morphologically, adult Triozidae are characteristic in a veination pattern in the forewing diverging from a single point, whereas adult Psyllidae are characteristic in having antennae with a second segment wider than the first and longer than the third [22]. To date, there has not been a comparative analysis among members of these two psyllid families at the mitogenome level.

While sequencing the genome of “*Ca*. L. solanacearum” from infected *B*. *cockerelli* DNA [23], a sequence contig containing an almost complete mitogenome was identified. The current report details the circularity and complete mitogenome of the *B*. *cockerelli*. Genes were annotated and comparative analyses were made between the mitogenome of *B*. *cockerelli* and those of the other three psyllids [12, 16, 19]. Efforts were made to evaluate *B*. *cockerelli* variations based on sequences of *cox*1 gene currently available in GenBank. Potential applications of the *B*. *cockerelli* mitogenome sequence in Psylloidea study were discussed.

## Materials and Methods

### Sample collection and DNA preparation

Adults of *B*. *cockerelli* were originally collected from the University of California South Coast Research and Extension Center in Irvine, California and maintained in a greenhouse at University of California at Riverside. DNA was extracted and purified from the individual psyllid using the DNeasy Blood and Tissue Kit (QIAGEN, Valencia, CA, USA). Briefly, individual insects were placed in microcentrifuge tubes containing 20 μL lysis buffer and homogenized by grinding with a plastic pestle (Kimble Chase, Vineland, NJ, USA). DNA isolation was carried out according to the manufacturer’s instructions. Sixty μL of DNA suspension were finally recovered from a spin column. DNA was amplified through illustra GenomiPhi V2 DNA Amplification Kit (GE Healthcare Inc., Waukesha, WI, USA).

### Mitogenome sequencing and assembling

The amplified DNA was sequenced using Illumina MiSeq format (Illumina, San Diego, CA, USA) and assembled *de novo* as described previously [23]. Illumina MiSeq was used because it generated a large volume of sequence data for high coverage *de novo* assembly. A single contig associated with mitogenome was identified by standalone BLASTn (version 2.2.30) [24] referenced to the complete mitogenome sequences of three Psylloidea species, NC_024577 (*P*. *sinica*), NC_027087 (*C*. *coccinea*) and NC_006157 (*P*. *venusta*) (Table 1), downloaded from GenBank database. Sequence of the contig was extracted using a Perl script. Coverage was calculated by mapping to the contig using paired reads of the MiSeq data by CLC Genomics Workbench 7.5 (CLC Bio, Denmark), with the following parameters: mismatch cost = 2, insertion cost = 3, deletion cost = 3, length fraction = 0.8, and similarity fraction = 0.9. The mitogenome circularity was verified by conventional PCR using the primers BC-mito-F (5’- GGT ATC TAA TCC TGG TTT AGC GC-3’) and BC-mito-R (5’-TTG TCT AAC ATT GGA GTG GGG-3’) designed by Primer3 [25] based on sequence from both end regions of the mitogenome contig. For PCR, reaction mixture (25 μL) contained: 20 ng of DNA template, 0.2 μM of each primer, 5 mM dNTP mixture, 2.5μL 10X buffer and 1 U of *TaKaRa Taq*HS enzyme. PCR amplification was: initial denaturation for 3 min at 95°C, followed by 35 cycles of denaturation for 45 s at 95°C, annealing for 30 s at 55°C, elongation for 2 min 30 s at 72°C, and a final extension step of 72°C for 10 min. Amplicons were electrophoresed on 1% agarose gel, collected and purified using NucleoSpin^®^ Gel and PCR Clean-up kit (QIAGEN, Valencia, USA). The purified DNA was sequenced using ABI 3130 DNA sequencer (ABI, Foster, CA, USA). The mitogenome of *B*. *cockerelli* was enclosed manually.

**Table 1:** General information of mitogenomes used in this study.

Superfamily	Family	Species	Accession number	Length of genome (bp)	Reference
Psylloidea	Triozidae	Bactericera cockerelli	**KU501214**	**15,220**	**This study**
		***Paratrioza sinica***	**NC_024577**	**14,863**	[19]
	Psyllidae	Cacopsylla coccinea	**NC_027087**	**14,832**	[16]
		Pachypsylla venusta	**NC_006157**	**14,711**	[12]
**Aphidoidea**	**Aphididae**	***Cervaphis quercus***	**NC_024926**	**15,272**	[15]
Aleyrodoidea	Aleyrodidae	***Aleurochiton aceris***	**NC_006160**	**15,388**	[12]

### Annotation and Sequence Analyses

PCGs of *B*. *cockerelli* were identified by ORF Finder software available at the website of NCBI (National Center for Biotechnology Information) with the invertebrate mitochondrial genetic codons. Gene boundaries were compared and confirmed with the annotated sequences of the three published psyllid mitogenomes (Table 1) using ClustalW as implemented in MEGA 6 [26]. tRNAs were predicted by their cloverleaf secondary structure using tRNAscan-SE 1.21 [27], ARWEN v1.2 [28] and MITOS [29]. Sequence tandem repeats were analyzed by Tandem Repeats Finder [30] (http://tandem.bu.edu/trf/trf.html). Sequence secondary structure was predicted by Mfold [31]. Nucleotide composition and codon usage were analyzed with MEGA 6 [26]. AT and GC-skew were calculated according to the formulae: AT skew = (fA−fT) / (fA + fT) and GC skew = (fG−fC) / (fG + fC), where fA, fT, fC and fG are proportions of each nucleotide [32]. Sliding window analyses were performed using DnaSP v5 [33]. Nucleotide diversities (Pi’s) among PCGs and rRNA genes were estimated by sliding window analyses (a 250 bp window in 25 bp overlapping steps) across the alignment of mitogenome sequences among the four Psylloidea members (Table 1). For *cox*1 locus evaluation and phylogenetic tree construction, available *B*. *cockerelli* sequences were downloaded from GenBank.

### Phylogenetic analysis

Phylogenetic relationships among the four Psylloidea members were analyzed based on mitogenome sequences along with two mitogenome sequences (NC_024926 for *Cervaphis quercus* and NC_006160 for *Aleurochiton aceris*) downloaded from GenBank as out groups (Table 1). The nucleotide sequences of 13 PCGs in the mitogenomes were collected and translated into amino acid sequences. The amino acid sequences were aligned by Clustal X [34] following the method of Jeyaprakash and Hoy [35]. Briefly, gap opening penalty = 35 and extension = 0.75 were set for pairwise sequence alignment; Gap opening penalty = 15 and extension = 0.3 were set for multiple sequence alignment; and the Gonnet protein weight matrix was used. The Gonnet matrix is an extension of the commonly used PAM matrix, but more suitable for large data set with more taxa or characters. With the help of a Perl script and referring to the amino acid sequences, the third nucleotide in each codon was removed in each gene sequence to minimize the effect of synonymous substitution leading to evaluation bias or skew [11]. For a cross-checking purpose, the procedure of Jeyaprakash and Hoy [35] was followed. Two software programs, PHYML 3.0 [36] for the maximum likelihood (ML) method and MrBayes (version: 3.2.5) [37] for the Bayesian inference (BI) method, were utilized to construct phylogenetic trees. For ML analyses, the optimal substitution model obtained from jModelTest [38] was used. Nodal support among branches was evaluated by bootstrap analysis with 100 replicates [39]. For BI analyses, two sets of four chains were allowed to run simultaneously for 1,000,000 generations, with sampling every 100 generations. After discarding the first 25% samples as burn-in, Bayesian posterior probability values were calculated in a consensus tree [40].

## Results

### Mitogenome organization of *B*. *cockerelli*

**Fig 1:**
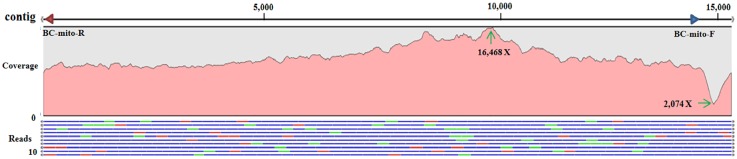
A schematic representation of *de novo* assembling and coverage estimate of the 15,263 bp mitogenome of *Bactericera cockerelli* using MiSeq data. In the top Contig line, the blue and red arrows represent the forward and reversed primers (BC-mito-F/BC-mito-R) used to verify the circularity of *B*. *cockerelli* mitogenome by PCR. Numbers are nucleotides in bp. In the Coverage section, the pink area represents nucleotide coverage with the highest of 16,468 X in *nad*6 and the lowest of 2,074 X in CR. In the Reads section, ten top read assemblings from MiSeq data were representatively shown with blue as pair reads, red as forward reads only and green as reversed reads only.

**Fig 2:**
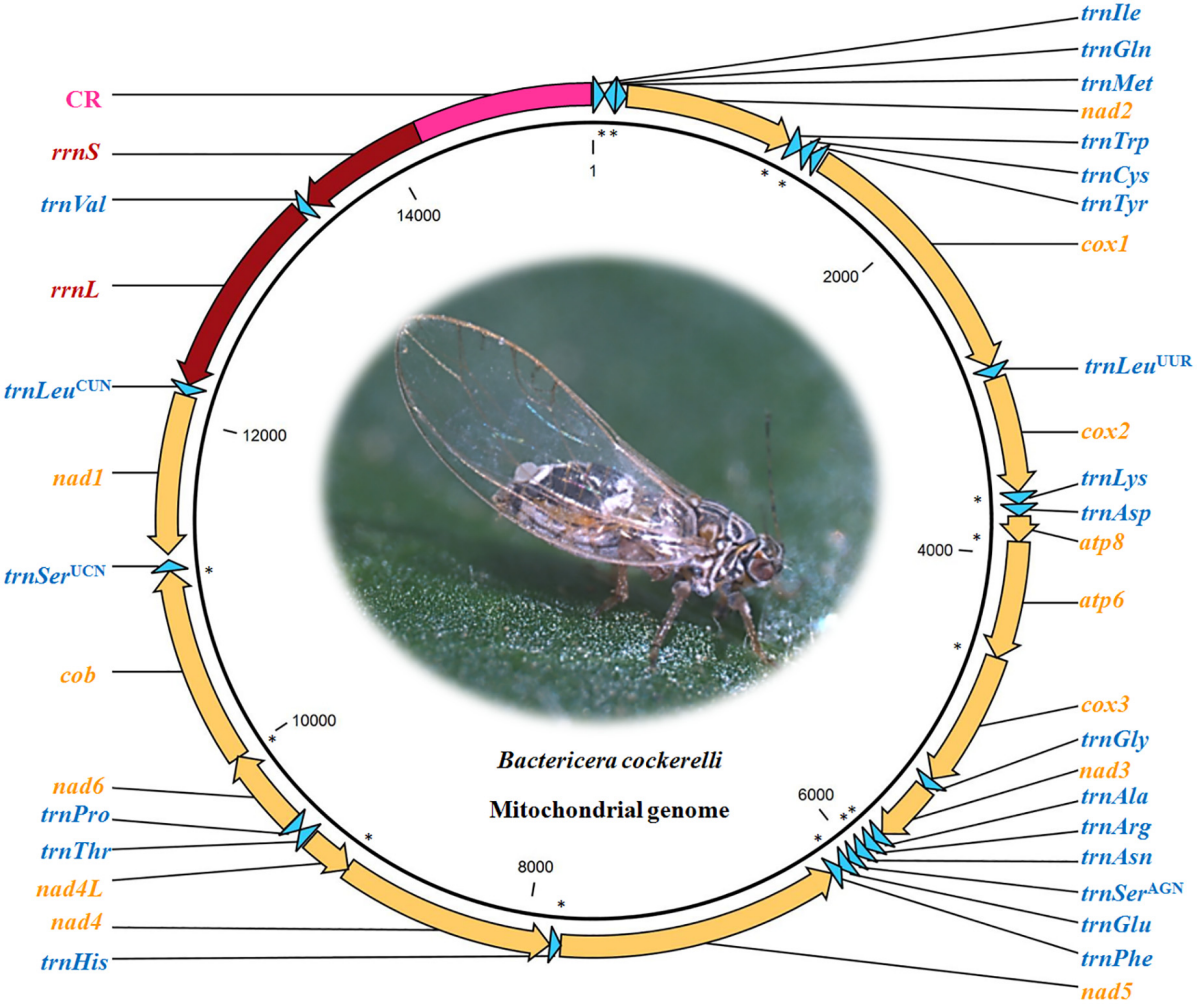
Map of the circular mitogenome (in bp) of *Bactericera cockerelli*. Majority strand (J-strand) is indicated by clockwise arrows, and minority strand (N-strand) in the opposite direction. *atp =* ATP synthase, *cob =* cytochrome oxidase b, *cox =* cytochrome oxidase c, *nad =* NADH dehydrogenase subunits, *rrnS =* small ribosomal RNA subunit, *rrnL* = large ribosomal RNA subunit, and CR = control region. A star “*” indicates the location of a gene overlap. Color codes: orange = protein coding genes, blue = tRNA genes, dark red = rRNA genes, and pink = control region.

From *de novo* assembly of MiSeq data, a single contig of 15,263 bp with a 43-bp duplication at both ends was identified by the mitogenome sequences of *P*. *sinica* (78%), C. coccinea (77%), and P. venusta (76%). Primer set BC-mito-F/BC-mito-R amplified a fragment of 1,285 bp, proving the circularity of the psyllid mitogenome (Figs 1 and 2). The mitogenome of *B*. *cockerelli* was determined to be 15,220 bp. Based on annotation, the *B*. *cockerelli* mitogenome included the entire set of 37 genes as those in the ancestral mitogenomes of insects [11, 41]. The average nucleotide coverage of the *B*. *cockerelli* mitogenome was 10,604 X, with the lowest (2,074 X) in control region and the highest (16,468 X) in the *nad6* gene. Twenty-three genes were on the majority strand (J-strand), and the other 14 genes on the minority strand (N-strand) (Fig 2). Sizes of all intergenic regions ranged from 3 to 31 bp with the exceptions of CR as discussed later. Gene overlaps were also observed: four between PCG and PCG (1–7 bp), seven between tRNA and tRNA (1–12 bp), and three between PCG and tRNA gene (2–3 bp) (Fig 2).

### Nucleotide compositions

The A+T content, GC-skew and AT-skew of the *B*. *cockerelli* and three other Psylloidea mitogenomes are listed in Table 2. The mitogenome of *B*. *cockerelli* was highly A+T biased with the control region being the highest (82.7%). Whole genome-wide, PCGs in N-strand PCGs were more AT-skewed (-0.248) than the J-strand (-0.084), i.e. PCGs in J-strand had nearly equal A and T. The tRNA genes in the N-strand was more GC-skewed (0.429) than those in the J-strand (0.022). No significant differences were found in both PCGs and tRNA genes between *B*. *cockerelli* and the other three psyllids. For rRNA genes, the A+T content of *B*. *cockerelli* and *P*. *sinica* (75.8% and 75.1%) were lower than those of *C*. *coccinea* and *P*. *venusta* (77.1% and 77.8).

**Table 2:** Nucleotide compositions in the genomes of *Bactericera cockerelli*, *Paratrioza sinica*, *Cacopsylla coccinea*, and *Pachypsylla venusta*.

Feature	Triozidae						Psyllidae					
	*Bactericera cockerelli*			*Paratrioza sinica*			*Cacopsylla coccinea*			*Pachypsylla venusta*		
	%A+T	AT-Skew	GC-Skew	%A+T	AT-Skew	GC-Skew	%A+T	AT-Skew	GC-Skew	%A+T	AT-Skew	GC-Skew
**Whole**	**74**	**0.034**	**-0.215**	**72**	**0.467**	**-0.249**	**72**	**0.596**	**-0.286**	**75**	**0.069**	**-0.256**
**PCG**	**72.6**	**-0.146**	**-0.044**	**70.2**	**-0.137**	**-0.047**	**70.4**	**-0.156**	**-0.084**	**73.7**	**-0.128**	**-0.074**
**PCG-J**	**72.3**	**-0.084**	**-0.213**	**70.3**	**-0.075**	**-0.240**	**70.3**	**-0.073**	**-0.298**	**72.7**	**-0.043**	**-0.265**
**PCG-N**	**73.1**	**-0.248**	**0.242**	**70.2**	**-0.241**	**0.272**	**70.7**	**-0.291**	**0.274**	**75.3**	**-0.266**	**0.277**
**tRNA genes**	**77.1**	**0.025**	**0.162**	**75.8**	**0.029**	**0.140**	**74.7**	**0.044**	**0.166**	**77.4**	**0.023**	**0.117**
**tRNA-J**	**76.9**	**0.056**	**0.022**	**76**	**0.059**	**0.023**	**74.3**	**0.090**	**-0.009**	**77.3**	**0.055**	**-0.005**
**tRNA-N**	**77.5**	**-0.032**	**0.429**	**75.4**	**-0.027**	**0.344**	**75.3**	**-0.037**	**0.484**	**77.7**	**-0.035**	**0.339**
**rRNA genes**	**75.8**	**-0.036**	**0.281**	**75.1**	**-0.079**	**0.328**	**77.1**	**-0.075**	**0.339**	**77.8**	**-0.075**	**0.316**
**rRNA-L**	**76**	**-0.042**	**0.333**	**75.1**	**-0.094**	**0.382**	**76.4**	**-0.079**	**0.353**	**78.8**	**-0.065**	**0.317**
**rRNA-S**	**75.4**	**-0.024**	**0.206**	**75.1**	**-0.056**	**0.25**	**78.0**	**-0.068**	**0.318**	**76.3**	**-0.089**	**0.315**
**Control region**	**82.7**	**-0.047**	**0.006**	**82.4**	**0.016**	**-0.187**	**78.8**	**-0.074**	**-0.113**	**83.9**	**0.013**	**-0.125**

### Codon usage of protein coding genes (PCGs)

**Fig 3:**
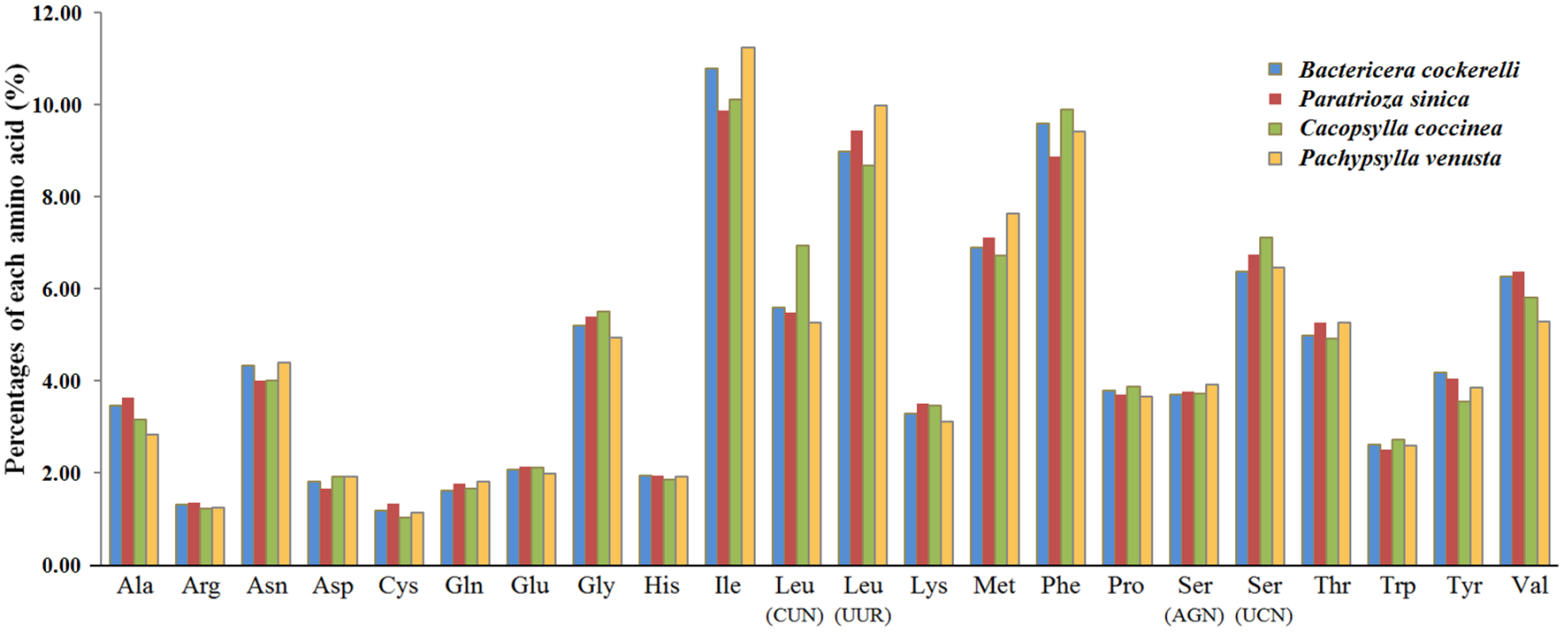
Percentages of amino acid usage in mitochondrial proteins of four Psylloidea species. Each amino acid is represented by its three letter abbreviation. Note that leucine and serine are each coded by two different genetic codons, and listed separately.

A total of 3,595 amino acids were coded for in the 13 PCGs of the *B*. *cockerelli* mitogenome. In terms of usage, the most frequently occurring amino acids were isoleucine (10.79%), phenylalanine (9.60%), and leucine (the UUR codon) (8.98%) (Fig 3). It should be noted that leucine and serine are each coded by two separate codon formats (CUN or UUR for leucine, and AGN or UCN for serine). The two codons formats for each were listed separately because they corresponded to different tRNAs [41]. Regardless, isoleucine, phenylalanine and leucine (UUR codon) also were in the top three amino acids in the mitogenome of the other three Psylloidea mitogenomes. On the other hand, arginine and cysteine were the least used amino acids in all four Psylloidea mitogenomes (1.31% and 1.20% for *B*. *cockerelli*, respectively) (Fig 3).

**Fig 4:**
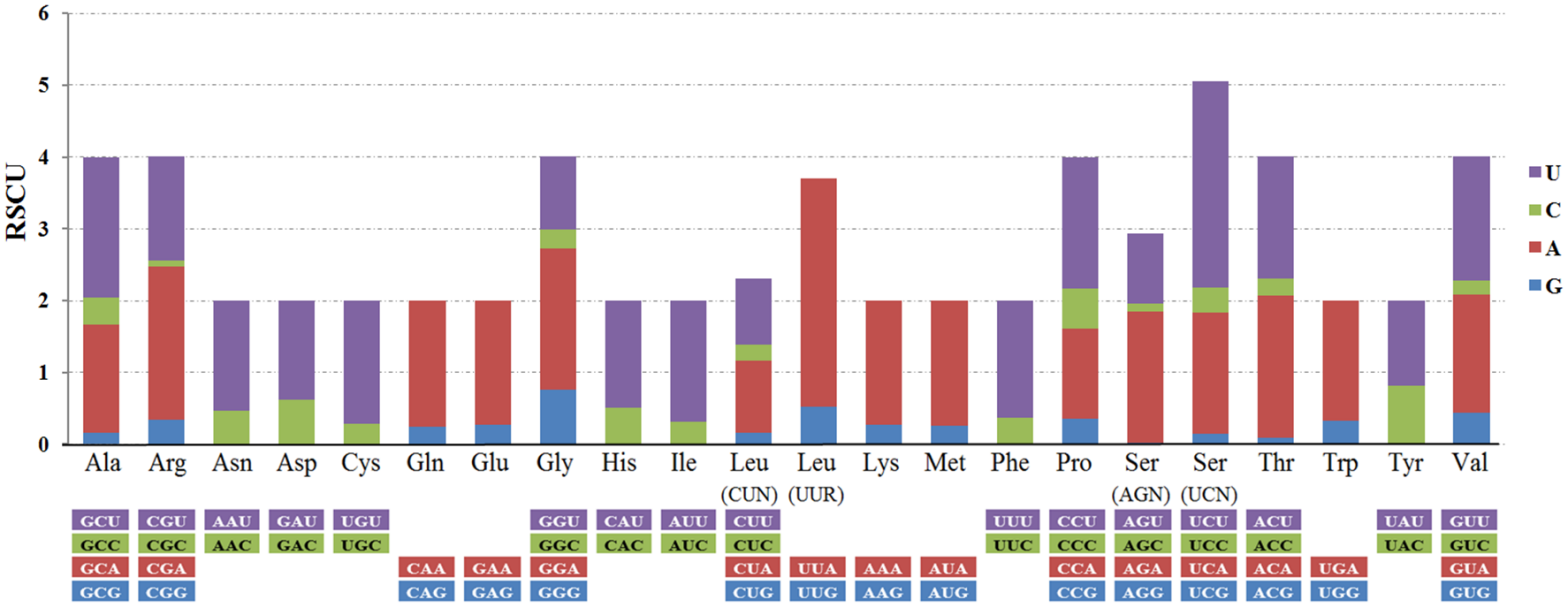
Relative synonymous codon usage (RSCU) in the mitogenome of *Bactericera cockerelli*. RSCU is defined as the ratio of the observed frequency of codons to the expected frequency given that all the synonymous codons for the same amino acids are used equally. Noted that the codons ending in U and A were the most frequent.

Relative synonymous codon usage (RSCU, defined as the ratio of the observed frequency of codons to the expected frequency given that all the synonymous codons for the same amino acids are used equally) in *B*. *cockerelli* were shown in Fig 4. Codons ending in U and A were the most frequent, which was associated with the high A+T content mitogenome. Similar RSCUs were also observed in the other three Psylloidea mitogenomes (S1 Table).

All 13 PCGs of *B*. *cockerelli* start with the typical “ATN” codon, similar to those of *P*. *sinica*, *C*. *coccinea* and *P*. *venusta*, with a lone exception for *nad5* in *P*. *sinica*, where “TTG” was used (Table 3) [12, 16, 19]. Four genes (*cox1*, *cox2*, *nad5* and *nad4*) of *B*. *cockerelli* also had incomplete stop codons with only a “T” present. Incomplete “T” stop codons occurred in all three other Psylloidea mitogenomes, albeit the number of genes with the incomplete stop codon varied between species (Table 3). With the exception of two genes (*cox2* and *nad1*), length of other 11 PCGs genes varied among the four Psylloidae mitogenomes (Table 3). The most variable gene was *nad4* with *C*. *coccinea* being 1,287 bp and *P*. *sinica*, *P*. *venusta* being 1,240 bp, i.e. a nine amino acid difference.

**Table 3:** Comparison of start and stop codons of protein coding genes (PCGs), and length of PCGs and rRNA genes among four mitogenomes of Psylloidea species.

Gene	*Bactericera cockerelli*			*Paratrioza sinica*			*Cacopsylla coccinea*			*Pachypsylla venusta*		
	Start codon	Stop codon	Length (bp)	Start codon	Stop codon	Length (bp)	Start codon	Stop codon	Length (bp)	Start codon	Stop codon	Length (bp)
***nad2***	**ATC**	**TAA**	**972**	**ATT**	**T**	**970**	**ATA**	**TAA**	**972**	**ATG**	**TAA**	**978**
***cox1***	**ATG**	**T**	**1,531**	**ATA**	**TAA**	**1,539**	**ATG**	**TAA**	**1,533**	**ATG**	**T**	**1,531**
***cox2***	**ATT**	**T**	**664**	**ATT**	**T**	**664**	**ATA**	**T**	**664**	**ATT**	**T**	**664**
***atp8***	**ATC**	**TAA**	**153**	**ATC**	**TAA**	**153**	**ATA**	**TAA**	**144**	**ATG**	**TAA**	**138**
***atp6***	**ATG**	**TAA**	**675**	**ATG**	**TA**	**674**	**ATG**	**TAA**	**675**	**ATG**	**TAA**	**675**
***cox3***	**ATG**	**TAA**	**783**	**ATG**	**TAA**	**783**	**ATG**	**TAA**	**780**	**ATG**	**T**	**781**
***nad3***	**ATT**	**TAA**	**351**	**ATT**	**T**	**349**	**ATA**	**T**	**352**	**ATA**	**TAA**	**354**
***nad5***	**ATT**	**T**	**1,627**	**TTG**	**T**	**1,624**	**ATT**	**T**	**1,621**	**ATG**	**T**	**1,618**
***nad4***	**ATG**	**T**	**1,243**	**ATG**	**T**	**1,240**	**ATG**	**TAG**	**1,287**	**ATG**	**T**	**1,240**
***nad4L***	**ATA**	**TAG**	**276**	**ATA**	**TAG**	**285**	**ATA**	**TAG**	**261**	**ATA**	**TAG**	**273**
***nad6***	**ATA**	**TAA**	**483**	**ATA**	**TAA**	**483**	**ATA**	**TAA**	**486**	**ATA**	**TAA**	**480**
***cob***	**ATA**	**TAG**	**1,143**	**ATA**	**T**	**1,126**	**ATA**	**TAG**	**1,146**	**ATA**	**TAG**	**1,137**
***nad1***	**ATA**	**TAA**	**915**	**ATA**	**TAA**	**915**	**ATA**	**TAG**	**915**	**ATT**	**TAA**	**915**
***rrnL***			**1,181**			**1,159**			**1,154**			**1,148**
***rrnS***			**800**			**802**			**773**			**765**

### Ribosomal RNA genes

The length of the two ribosomal RNA gene *rrnL*(16S) and *rrnS* (12S) in *B*. *cockerelli* are 1,181 bp and 800 bp, respectively. The *rrnL* of *B*. *cockerelli* is over 20 bp longer than other three Psylloidea species (Table 3). BLASTn searches using *rrnL* of *B*. *cockerelli* as query against complete mitogenome database in GenBank identified three top hits: *P*. *sinica* (Query cover: 92%; identity: 84%), *C*. *coccinea* (Query cover: 89%; identity: 79%), and *P*. *venusta* (Query cover: 55%; identity: 82%). BLASTn searches of the *rrnS* shows that there is a fairly homologous core with *P*. *sinica* (Query cover: 97%; identity: 85%), *C*. *coccinea* (Query cover: 97%; identity: 78%) and *P*. *venusta* (Query cover: 96%; identity: 78%).

### Diversity of protein coding genes (PCGs) and rRNA genes within Psylloidea

**Fig 5:**
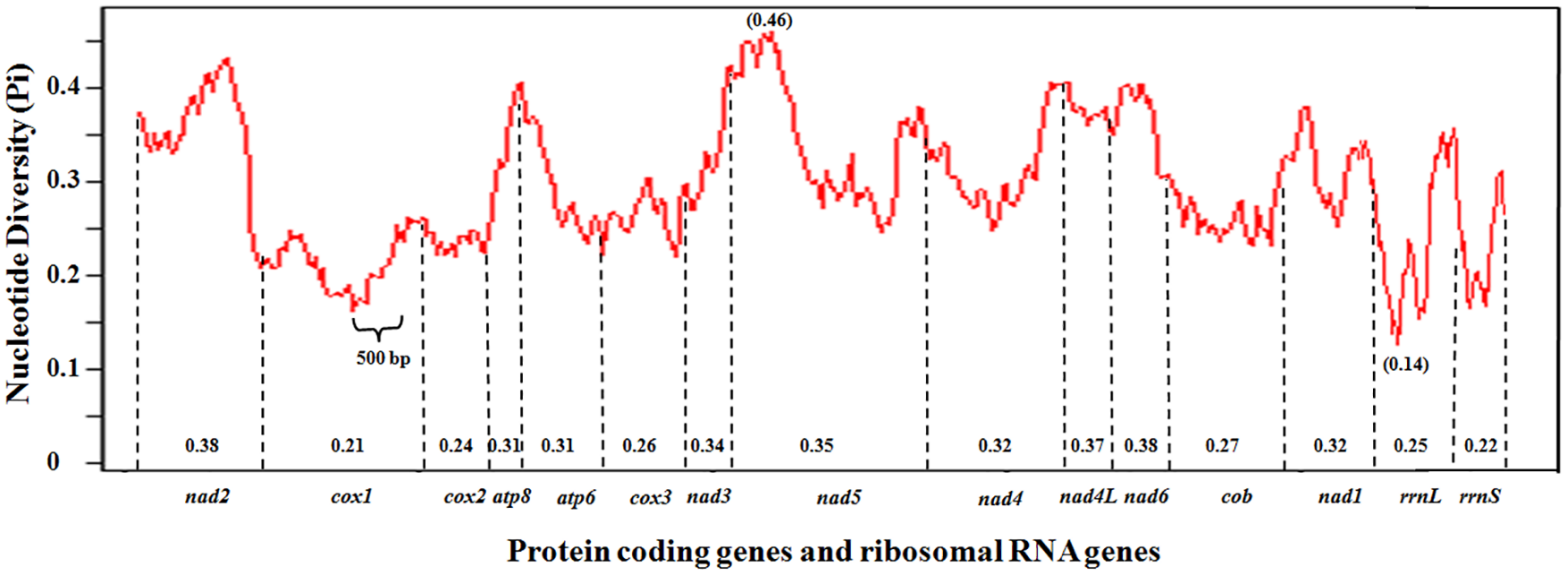
Sliding window analyses of protein coding genes and ribosomal RNA genes among Psylloidea mitogenomes. The graph shows the change of nucleotide diversity (Pi) in a sliding window of 250 bp with the step size of 25 bp. The average of Pi values of each gene are presented at the bottom calculated using DnaSP v5 [33] software. Note the location of the 500-bp region in *cox1* that was used for *Bactericera cockerelli* haplotyping.

Nucleotide diversity of the 13 PCGs among the four mitogenomes of Psyllidae species is shown in Fig 5. On average, genes *nad2* (Pi = 0.38), *nad4L* (Pi = 0.37) and *nad6* (Pi = 0.38) displayed the highest variability. However, the most variable region (window) was in *nad5* (Pi = 0.46) despite of its mean value of 0.35. On the other hand, *cox1* (Pi = 0.21), *cox2* (Pi = 0.24), and *cox3* (Pi = 0.26) were the most conserved PCGs. Both *rrnL* (Pi = 0.25) *and rrnS* (Pi = 0.22) were also highly conserved.

**Fig 6:**
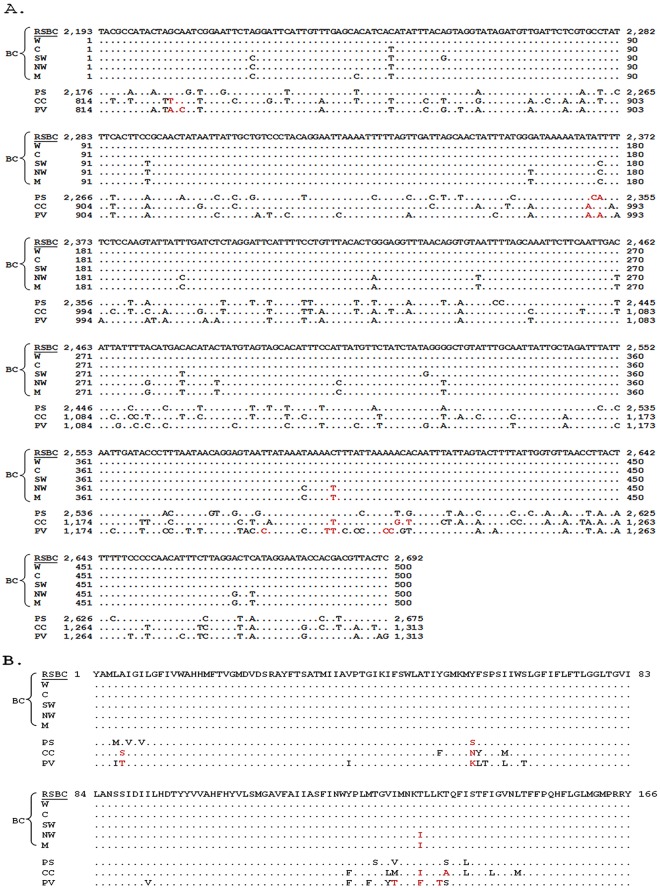
Alignment of a 500-bp nucleotide sequence in *cox1* (A) and the corresponding amino acid sequences (B) among four Psylloidea mitogenomes including *Bactericera cockerelli* haplotypes and other sequences. **A:** BC, *B*. *cockerelli*. RSBC, the *B*. *cockerelli* used in the study. W: Western haplotype (JQ708095 and AY971885). C: Central haplotype (JQ708094, FJ175374, EF372597 and AY971888). SW: Southwestern haplotype (KC305359). NW: Northwestern haplotype (JQ708093, KR534770, KR534769, KR534767, KR534766 and KR534765). M, KR534768. PS, *Paratrioza sinica*. CC, *Cacopsylla coccinea*. PV, *Pachypsylla venusta*. Nucleotide variations at non-third codon position and the corresponding amino acids are in red.

Fig 6A shows the sequence alignment of a 500 bp regions in the *cox1* among the four Psylloidea species. This was the region used for establishment of *B*. *cockerelli* haplotypes [9, 10] and the California psyllid (RSTM) in this study fit in the Western haplotype. Currently, there are 14 *cox1* sequences of *B*. *cockerelli* deposit in GenBank (release 211.0). By focusing the sequences corresponding to the 500-bp region, all *cox1* sequences fit into one of the four haplotypes with the exception two sequences represented by AY971885, designated as M, that were identical to the Northwestern haplotype with a SNP (Fig 6A). Regardless of the numbers of SNPs (from 1 to 19), when the corresponding amino acid sequences were compared, all *cox1* sequences from different psyllids shared a 100% identity with the only exception being that of the Northwestern haplotype and sequence M, which had isoleucine (I) in the place of threonine (T) at position 134 (Fig 6B). There were six *cox1* sequences of the Northwestern haplotype thus far submitted to Genbank. Isoleucine has a hydrophobic side chain, whereas threonine has a polar uncharged side chain. Therefore, this amino acid switch could have an impact on the structure of the *cox1* protein.

As expected, significantly more variations were found among the four Psylloidea species at the nucleotide level (Fig 6A). Total similarity (BLASTn identity) between *B*. *cockerelli* and *P*. *sinica* (Triozidae) was 85%, higher than that between *B*. *cockerelli* and the two Psyllidae members [C. coccinea (81%) and P. venusta (82%)]. Accordingly, there were 8 to 23 substitutions that occurred at the amino acid level (Fig 6B).

### Transfer RNA genes

**Fig 7:**
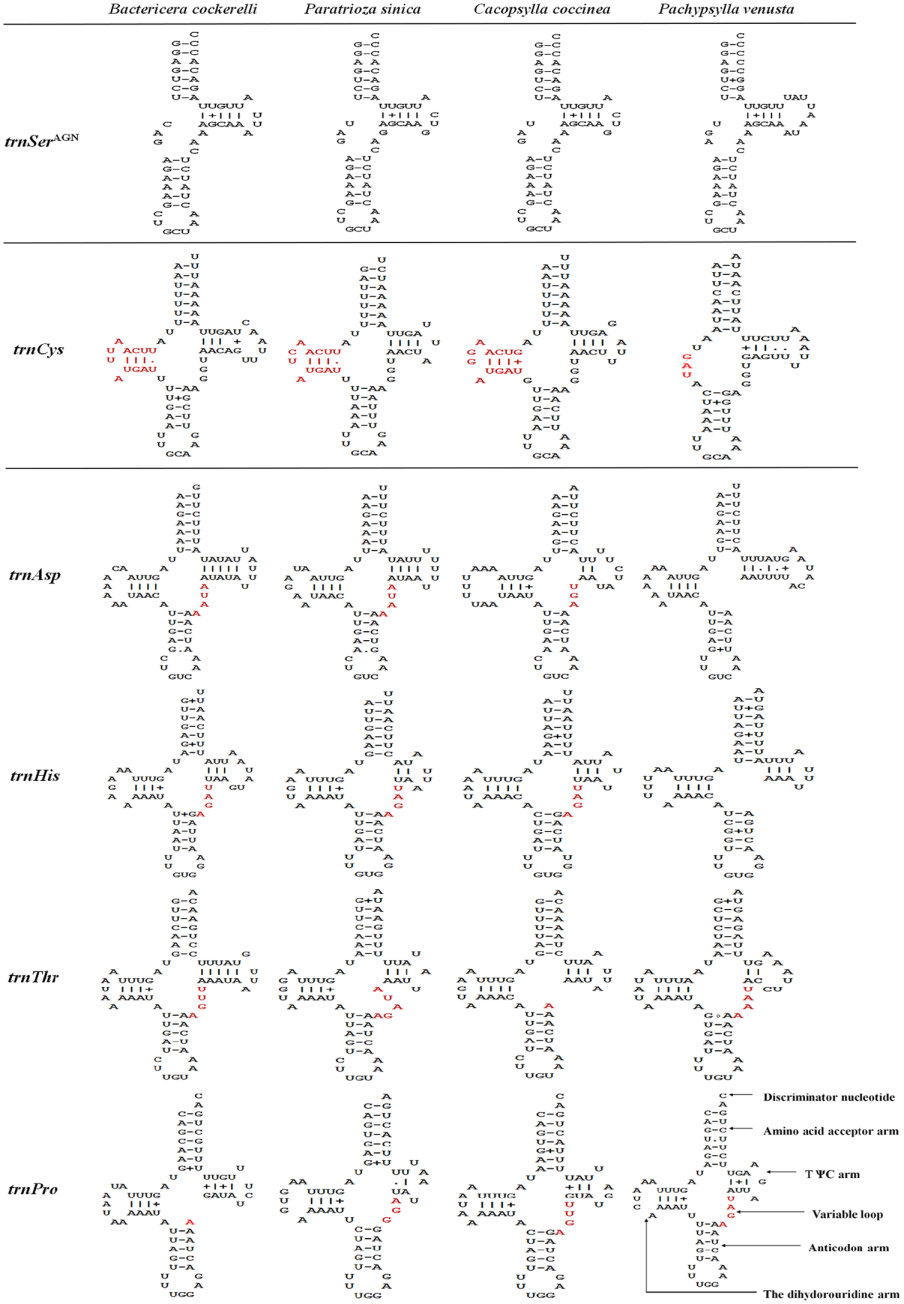
Secondary structures of the three types of tRNA genes identified in the mitogenome of four Psylloidea species. Bar “-”, Watson-Crick base pairing. Others are canonical base parings in tRNA: Plus sign “+”, a paring between G and U; Dot “•” A paring between U and U; And Hollow dot “◦”, a paring between A and G. Bases highlighted in red indicated the different structure among the four Psylloidea members.

Twenty-two tRNA genes ranging in size from 54 to 75 bp were identified in the mitogenome of *B*. *cockerelli*. They all had standard cloverleaf structure, which was a typical feature of metazoan mitogenomes [42] with the exception of *trnSer*^AGN^ with a missing dihydorouridine (DHU) arm (Fig 7). In conducting a comparison among the four Psylloidea species, two differences were noted (Fig 7): 1) the incomplete DHU arm in *trnCys* (*P*. *venusta*); and 2) the loss of the incomplete variable loop in *trnAsp* and *trnHis* (*P*. *venusta*), *trnThr* (*C*. *coccinea*), and *trnPro* (*B*. *cockerelli*).

### Control region

**Fig 8:**
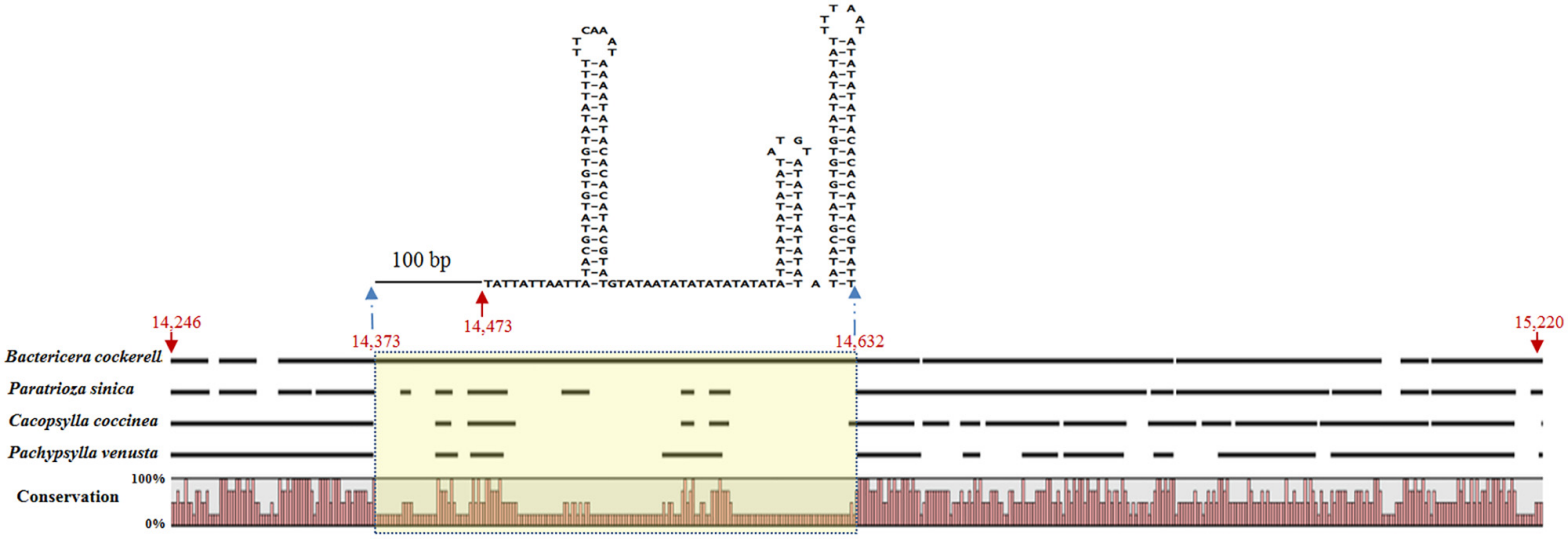
Schematic nucleotide alignments of the Control Regions (CRs) among four Psylloidea mitogenomes and the unique region in *Bactericera cockerelli* with a predicted secondary structure.

The length of the *B*. *cockerelli* control region is 975 bp, longer than that of *C*. *coccinea* (671 bp), *P*. *venusta* (597 bp) and *P*. *sinica* (700 bp) [12, 16, 19]. To further confirm the length consistency of *B*. *cockerelli*, ten adults were selected for conventional PCR using primer set BC-mito-F/BC-mito-R, resulting in the same amplified DNA fragment. There were no tandem repeats with a repeat unit size>2 bp detected in *B*. *cockerelli*, *C*. *coccinea*, and *P*. *venusta*. For *P*. *sinica*, 3.2 tandem repeats with the unit size of 14 and 85% unit similarity were identified. Alignment of the control regions from four Psylloidea mitogenomes revealed an insertion sequence of 260 bp (from position 14,373 to position 14,632) in the *B*. *cockerelli* mitogenome (Fig 8). This region could be folded into a three stem-loop secondary structure (Fig 8).

### Phylogenetic relationships

**Fig 9:**
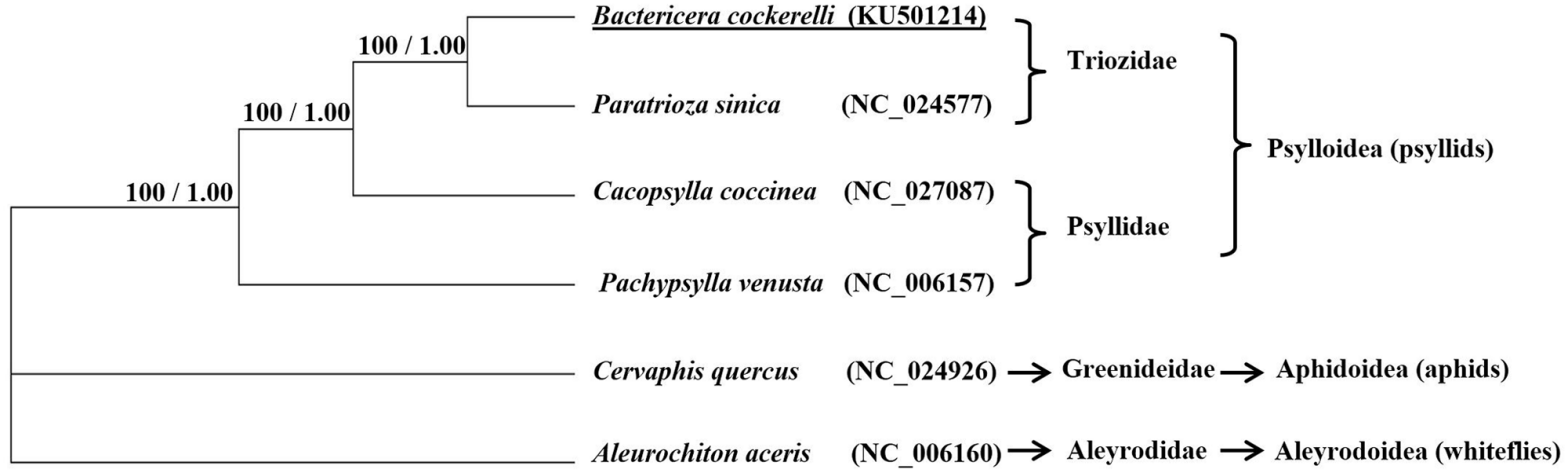
Phylogenetic relationship of four members of Psylloidea based on 13 protein coding gene sequence in their mitogenomes. Numbers at the nodes are bootstrap values of maximum likelihood method / posterior probabilities of Bayesian inference method. The mitogenomes of *Cervaphis quercus* and *Aleurochiton aceris* were used as outgroup. Numbers in the brackets represent the GenBank accession numbers.

As shown in Fig 9, from both ML and BI methods, *B*. *cockerelli* was grouped with *P*. *sinica*, another member of the family Triozidae, and separated to *C*. *coccinea* and *P*. *venusta*, both in the Psyllidae. Both the bootstrap value (100%) in ML analyses and Bayesian posterior probabilities (1.00) in BI analyses indicated the high level of reliability of the analyses. The phylogenetic analyses of the Psylloidea indicated a close relationship with *Cervaphis quercus*, which belongs to Aphidoidea, even though that latter was comprised of partial mitogenome, albeit one with high reliability [12, 43].

## Discussion

Nucleotide sequences from mitochondria have been successfully used to evaluate population variations of *B*. *cockerelli* albeit only 500 bp region of the 1,531 bp *cox1* gene were considered [8–10] (Table 3). As shown in Fig 6B and also discussed by Swisher *et al* [10], all currently identified SNPs are in the third position of the codons. In the context of this study (Fig 6A and 6B), the nucleotide substitutions at the third position nucleotide would not affect the gene function, suggesting the substitutions or at least some of the substitutions may not be stable and undermining its use for haplotyping. For example, sequence M (KR534768) is identical to the Northwestern haplotype but a SNP (Fig 6A). Likewise, the sequences for Central and Western haplotypes differ only by a SNP (Fig 6A). A natural question that arises is whether a SNP in a single gene should be cause for considering defining a novel haplotype. In this consideration, it deems to be necessary to further evaluate the population of *B*. *cockerelli* based on more sequence variations. The mitogenome sequence published here will potentially be a guide that can be used for selecting other genes for establishing haplotypes. If there are additional suitable regions for haplotype definition, additional data can be collected to verify existing schemes to separate psyllid populations. There likely is not a requirement to sequence the mitogenomes of all defined haplotypes of *B*. *cockerelli*, as the careful selection of both conservative and highly variable regions together should yield robust data to separate populations. That said, NGS techniques are becoming more and more accessible, and in the near future, NGS-based mitogenome sequencing according to the methods employed herein could be used to facilitate sequencing all or the majority of proposed psyllid haplotypes.

Thao et al. published a 3,077 bp sequence of the *B*. *cockerelli* mitogenome (AY601890) for a genome organization study of three Sternorrhyncha members (whiteflies, aphids and psyllids) [12]. The sequence covered two complete PCGs, *nad1* and *rrnL*. An analysis on *rrnL* sequence revealed SNPs among the four *B*. *cockerelli* haplotypes [44]. Interestingly, there was also only one SNP between the Western and Central haplotypes. Furthermore, AY601890 was not identical to any of the four haplotypes [44]. Interestingly, there was also only one SNP between the Westernand Central haplotypes. Furthermore, AY601890 was not identical to any of the four haplotypes [44]. Powell *et al* [45] also explored the use of AY601890 but they detected no variations among the psyllid samples collected from Colorado, Washington and Texas. The complete mitogenome sequence presented in this study provides feasible access to the sequence of every mitochondrial gene for future *B*. *cockerelli* -SNP analyses. For example, 31 SNPs were identified by comparison of this mitogenome sequence with AY601890 (S1 Fig) outside the *cox1* gene. All *B*. *cockerelli* haplotypes/biotypes in future will be screened for these newly discovered SNPs.

In view with gene diversity among the four Psyllidea members, the most conserved gene is *cox1* with the mean Pi value of 0.21 (Fig 5). The large number of SNPs among the four Psylloidea members suggests that sequence in this region could be effective for differentiation of the four species. Referenced to the *B*. *cockerelli* mitogenome sequence, the 500 bp region used for existing biotyping / haplotyping studies [8–10] was located in the most conserved part of the *cox1* gene (Fig 5). As such, populations could likely be described with establishment of SNPs as observed in that region first. However, other SNPs in more variable portions could reveal additional haplotypes, as it is easy to speculate that if the more variable portion of the *cox1* gene were used, then, more SNPs could be found.

In addition to *cox1*, other genes also have potential to be used for population analyses. For example, *nad1* had been successfully used to analyze the phylogeny of aphid parasitoids (Hymenoptera: Braconidae: Aphidiinae) [46]. Interestingly, 14 SNPs were revealed by the comparison between the *nad1* sequence from this study to that of AY601890 [12] (S1 Fig) Inferred by the data at the family/genus level, *nad4* sequence has a higher Pi value of 0.32 than that of *cox1* (Fig 5). Interestingly, *nad4* shows a sequence that varies up to 47 bp among the four Psylloidea members (Table 3). Therefore, *nad4* could be candidate for future evaluation for diversity study among *B*. *cockerelli* members. Another sequence to consider is that of *rrnL*, which was tested with limited number of samples [44], and suggested by the inter-family/genus analysis (Fig 5) that *rrnL* had a 25-bp region with the lowest Pi value of 0.14. Sequence of this gene had been used to explore phylogenetics of Dictyoptera insects [47].

Due to existing technical difficulty, only a limited number of insect mitogenomes have been sequenced The fact that only four mitogenome sequences were used for phylogentic evaluation of Psylloidea could be considered to lack robustness across the wide variety of insects for whom also could have been included in this study. However, the clustering of the two Triozidae members and the two Psylllidae members suggests that mitogenome-based grouping is in line with current morphology-based taxonomy of two sub-families in Psyllidea (Fig 9), and it is likely adding more members would further confirm this. For *B*. *cockerelli*, the complete mitogenome from this study could provide a template for primer design to study gene or even complete mitogenome sequence variations through the traditional PCR approach [12–16] although the advancement of NGS technology and the drop of sequence cost are expected to be the main technique for future whole mitogenome sequencing.

## Conclusions

The present study presents the first complete mitogenome of *B*. *cockerelli*. The mitogenome contains 37 genes (13 PCGs, 2 rRNA and 22 tRNA) and a control region (CR). The gene order of the mitogenome matches with that of the common ancestral insect mitogenome. However, sequence variations existed when compared with other mitogenomes of three other Psylloidea members, mostly in the form of SNPs and small nucleotide insertions / deletions, particularly the CR. Knowledge gained from this study will allow future phylogenetic studies to proceed, and improved understanding of the population genetics of psyllids should help development and testing of new management options.

## Supporting Information

S1 Table A: Codon usage of protein coding genes of *Bactericera cockerelli*, B: *Paratrioza sinica*, C: *Cacopsylla coccinea*, and D: *Pachypsylla venusta*. (DOCX)

S1 Fig Alignment between AY601890 (3,077 bp, Thao et al., 2004) and the corresponding region in the mitogenome sequence of *Bactericera cockerelli* (KU501214). *cob =* cytochrome oxidase b, *nad =* NADH dehydrogenase subunits, *rrnL* = large ribosomal RNA subunit *rrnS =* small ribosomal RNA subunit. Sequence codes: yellow = protein coding genes, green = rRNA genes, and underlined = tRNA genes. Identical nucleotides are represented by dot “.”, and single nucleotide polimorphisms are indicated by letters in red.
(TIF)
