# Supplementary material for: The Complete Mitochondrial Genome Sequence of Bactericera cockerelli and Comparison with Three Other Psylloidea Species
Source: PLoS One. 2016 May 26;11(5):e0155318. doi: 10.1371/journal.pone.0155318 (PMC4881912; doi:10.1371/journal.pone.0155318)
Supplement: S1 Table — (DOCX) [file pone.0155318.s001.docx]

**Table S1A. Codon usage of protein coding genes** **of *Bactericera cockerelli*.**

| Codon | Count | RSCU | Codon | Count | RSCU | Codon | Count | RSCU | Codon | Count | RSCU |
| --- | --- | --- | --- | --- | --- | --- | --- | --- | --- | --- | --- |
| UUU(F) | 282 | 1.63 | UCU(S) | 130 | 2.87 | UAU(Y) | 89 | 1.19 | UGU(C) | 37 | 1.72 |
| UUC(F) | 63 | 0.37 | UCC(S) | 16 | 0.35 | UAC(Y) | 61 | 0.81 | UGC(C) | 6 | 0.28 |
| UUA(L) | 278 | 3.18 | UCA(S) | 76 | 1.68 | UAA(*) | 0 | 0 | UGA(W) | 79 | 1.68 |
| UUG(L) | 45 | 0.52 | UCG(S) | 7 | 0.15 | UAG(*) | 0 | 0 | UGG(W) | 15 | 0.32 |
| CUU(L) | 81 | 0.93 | CCU(P) | 62 | 1.82 | CAU(H) | 52 | 1.49 | CGU(R) | 17 | 1.45 |
| CUC(L) | 19 | 0.22 | CCC(P) | 19 | 0.56 | CAC(H) | 18 | 0.51 | CGC(R) | 1 | 0.09 |
| CUA(L) | 87 | 1 | CCA(P) | 43 | 1.26 | CAA(Q) | 51 | 1.76 | CGA(R) | 25 | 2.13 |
| CUG(L) | 14 | 0.16 | CCG(P) | 12 | 0.35 | CAG(Q) | 7 | 0.24 | CGG(R) | 4 | 0.34 |
| AUU(I) | 328 | 1.69 | ACU(T) | 76 | 1.7 | AAU(N) | 120 | 1.54 | AGU(S) | 44 | 0.97 |
| AUC(I) | 60 | 0.31 | ACC(T) | 11 | 0.25 | AAC(N) | 36 | 0.46 | AGC(S) | 5 | 0.11 |
| AUA(M) | 217 | 1.75 | ACA(T) | 88 | 1.97 | AAA(K) | 102 | 1.73 | AGA(S) | 83 | 1.83 |
| AUG(M) | 31 | 0.25 | ACG(T) | 4 | 0.09 | AAG(K) | 16 | 0.27 | AGG(S) | 1 | 0.02 |
| GUU(V) | 97 | 1.72 | GCU(A) | 61 | 1.95 | GAU(D) | 45 | 1.38 | GGU(G) | 48 | 1.03 |
| GUC(V) | 11 | 0.2 | GCC(A) | 12 | 0.38 | GAC(D) | 20 | 0.62 | GGC(G) | 12 | 0.26 |
| GUA(V) | 92 | 1.64 | GCA(A) | 47 | 1.5 | GAA(E) | 65 | 1.73 | GGA(G) | 92 | 1.97 |
| GUG(V) | 25 | 0.44 | GCG(A) | 5 | 0.16 | GAG(E) | 10 | 0.27 | GGG(G) | 35 | 0.75 |

**Table S1B. Codon usage of protein coding genes** **of *Paratrioza sinica*.**

| Codon | Count | RSCU | Codon | Count | RSCU | Codon | Count | RSCU | Codon | Count | RSCU |
| --- | --- | --- | --- | --- | --- | --- | --- | --- | --- | --- | --- |
| UUU(F) | 248 | 1.55 | UCU(S) | 112 | 2.38 | UAU(Y) | 98 | 1.34 | UGU(C) | 38 | 1.58 |
| UUC(F) | 71 | 0.45 | UCC(S) | 26 | 0.55 | UAC(Y) | 48 | 0.66 | UGC(C) | 10 | 0.42 |
| UUA(L) | 284 | 3.18 | UCA(S) | 92 | 1.95 | UAA(*) | 0 | 0 | UGA(W) | 70 | 1.56 |
| UUG(L) | 55 | 0.62 | UCG(S) | 12 | 0.25 | UAG(*) | 0 | 0 | UGG(W) | 20 | 0.44 |
| CUU(L) | 75 | 0.84 | CCU(P) | 62 | 1.86 | CAU(H) | 45 | 1.29 | CGU(R) | 17 | 1.39 |
| CUC(L) | 27 | 0.3 | CCC(P) | 37 | 1.11 | CAC(H) | 25 | 0.71 | CGC(R) | 4 | 0.33 |
| CUA(L) | 81 | 0.91 | CCA(P) | 29 | 0.87 | CAA(Q) | 52 | 1.63 | CGA(R) | 20 | 1.63 |
| CUG(L) | 14 | 0.16 | CCG(P) | 5 | 0.15 | CAG(Q) | 12 | 0.38 | CGG(R) | 8 | 0.65 |
| AUU(I) | 279 | 1.57 | ACU(T) | 91 | 1.93 | AAU(N) | 103 | 1.43 | AGU(S) | 48 | 1.02 |
| AUC(I) | 76 | 0.43 | ACC(T) | 23 | 0.49 | AAC(N) | 41 | 0.57 | AGC(S) | 13 | 0.28 |
| AUA(M) | 208 | 1.63 | ACA(T) | 70 | 1.48 | AAA(K) | 105 | 1.67 | AGA(S) | 68 | 1.44 |
| AUG(M) | 48 | 0.38 | ACG(T) | 5 | 0.11 | AAG(K) | 21 | 0.33 | AGG(S) | 6 | 0.13 |
| GUU(V) | 87 | 1.52 | GCU(A) | 56 | 1.71 | GAU(D) | 36 | 1.2 | GGU(G) | 31 | 0.64 |
| GUC(V) | 16 | 0.28 | GCC(A) | 19 | 0.58 | GAC(D) | 24 | 0.8 | GGC(G) | 11 | 0.23 |
| GUA(V) | 98 | 1.71 | GCA(A) | 48 | 1.47 | GAA(E) | 60 | 1.56 | GGA(G) | 90 | 1.86 |
| GUG(V) | 28 | 0.49 | GCG(A) | 8 | 0.24 | GAG(E) | 17 | 0.44 | GGG(G) | 62 | 1.28 |

**Table S1C. Codon usage of protein coding genes** **of *Cacopsylla coccinea*.**

| Codon | Count | RSCU | Codon | Count | RSCU | Codon | Count | RSCU | Codon | Count | RSCU |
| --- | --- | --- | --- | --- | --- | --- | --- | --- | --- | --- | --- |
| UUU(F) | 281 | 1.58 | UCU(S) | 117 | 2.4 | UAU(Y) | 87 | 1.36 | UGU(C) | 28 | 1.51 |
| UUC(F) | 75 | 0.42 | UCC(S) | 40 | 0.82 | UAC(Y) | 41 | 0.64 | UGC(C) | 9 | 0.49 |
| UUA(L) | 271 | 2.89 | UCA(S) | 92 | 1.89 | UAA(*) | 0 | 0 | UGA(W) | 84 | 1.71 |
| UUG(L) | 42 | 0.45 | UCG(S) | 7 | 0.14 | UAG(*) | 0 | 0 | UGG(W) | 14 | 0.29 |
| CUU(L) | 104 | 1.11 | CCU(P) | 62 | 1.77 | CAU(H) | 48 | 1.43 | CGU(R) | 10 | 0.91 |
| CUC(L) | 25 | 0.27 | CCC(P) | 39 | 1.11 | CAC(H) | 19 | 0.57 | CGC(R) | 4 | 0.36 |
| CUA(L) | 112 | 1.19 | CCA(P) | 31 | 0.89 | CAA(Q) | 55 | 1.83 | CGA(R) | 25 | 2.27 |
| CUG(L) | 9 | 0.1 | CCG(P) | 8 | 0.23 | CAG(Q) | 5 | 0.17 | CGG(R) | 5 | 0.45 |
| AUU(I) | 287 | 1.58 | ACU(T) | 87 | 1.97 | AAU(N) | 99 | 1.38 | AGU(S) | 52 | 1.07 |
| AUC(I) | 77 | 0.42 | ACC(T) | 24 | 0.54 | AAC(N) | 45 | 0.63 | AGC(S) | 10 | 0.21 |
| AUA(M) | 202 | 1.67 | ACA(T) | 61 | 1.38 | AAA(K) | 102 | 1.63 | AGA(S) | 69 | 1.42 |
| AUG(M) | 40 | 0.33 | ACG(T) | 5 | 0.11 | AAG(K) | 23 | 0.37 | AGG(S) | 3 | 0.06 |
| GUU(V) | 90 | 1.72 | GCU(A) | 52 | 1.82 | GAU(D) | 49 | 1.42 | GGU(G) | 22 | 0.44 |
| GUC(V) | 25 | 0.48 | GCC(A) | 19 | 0.67 | GAC(D) | 20 | 0.58 | GGC(G) | 11 | 0.22 |
| GUA(V) | 65 | 1.24 | GCA(A) | 40 | 1.4 | GAA(E) | 57 | 1.5 | GGA(G) | 92 | 1.86 |
| GUG(V) | 29 | 0.56 | GCG(A) | 3 | 0.11 | GAG(E) | 19 | 0.5 | GGG(G) | 73 | 1.47 |

**Table S1D. Codon usage of protein coding genes** **of *Pachypsylla venusta*.**

| Codon | Count | RSCU | Codon | Count | RSCU | Codon | Count | RSCU | Codon | Count | RSCU |
| --- | --- | --- | --- | --- | --- | --- | --- | --- | --- | --- | --- |
| UUU(F) | 280 | 1.66 | UCU(S) | 114 | 2.45 | UAU(Y) | 94 | 1.36 | UGU(C) | 35 | 1.71 |
| UUC(F) | 58 | 0.34 | UCC(S) | 22 | 0.47 | UAC(Y) | 44 | 0.64 | UGC(C) | 6 | 0.29 |
| UUA(L) | 309 | 3.39 | UCA(S) | 92 | 1.97 | UAA(*) | 0 | 0 | UGA(W) | 83 | 1.78 |
| UUG(L) | 49 | 0.54 | UCG(S) | 4 | 0.09 | UAG(*) | 0 | 0 | UGG(W) | 10 | 0.22 |
| CUU(L) | 71 | 0.78 | CCU(P) | 61 | 1.86 | CAU(H) | 45 | 1.3 | CGU(R) | 18 | 1.6 |
| CUC(L) | 17 | 0.19 | CCC(P) | 25 | 0.76 | CAC(H) | 24 | 0.7 | CGC(R) | 1 | 0.09 |
| CUA(L) | 93 | 1.02 | CCA(P) | 43 | 1.31 | CAA(Q) | 56 | 1.72 | CGA(R) | 20 | 1.78 |
| CUG(L) | 8 | 0.09 | CCG(P) | 2 | 0.06 | CAG(Q) | 9 | 0.28 | CGG(R) | 6 | 0.53 |
| AUU(I) | 349 | 1.73 | ACU(T) | 70 | 1.48 | AAU(N) | 108 | 1.37 | AGU(S) | 47 | 1.01 |
| AUC(I) | 54 | 0.27 | ACC(T) | 21 | 0.44 | AAC(N) | 50 | 0.63 | AGC(S) | 8 | 0.17 |
| AUA(M) | 235 | 1.72 | ACA(T) | 92 | 1.95 | AAA(K) | 93 | 1.66 | AGA(S) | 83 | 1.78 |
| AUG(M) | 39 | 0.28 | ACG(T) | 6 | 0.13 | AAG(K) | 19 | 0.34 | AGG(S) | 3 | 0.06 |
| GUU(V) | 88 | 1.85 | GCU(A) | 50 | 1.96 | GAU(D) | 52 | 1.51 | GGU(G) | 38 | 0.86 |
| GUC(V) | 6 | 0.13 | GCC(A) | 9 | 0.35 | GAC(D) | 17 | 0.49 | GGC(G) | 3 | 0.07 |
| GUA(V) | 80 | 1.68 | GCA(A) | 42 | 1.65 | GAA(E) | 63 | 1.77 | GGA(G) | 112 | 2.53 |
| GUG(V) | 16 | 0.34 | GCG(A) | 1 | 0.04 | GAG(E) | 8 | 0.23 | GGG(G) | 24 | 0.54 |
