# Supplementary figures and images for: The Complete Mitochondrial Genome Sequence of Bactericera cockerelli and Comparison with Three Other Psylloidea Species
Source: PLoS One. 2016 May 26;11(5):e0155318. doi: 10.1371/journal.pone.0155318 (PMC4881912; doi:10.1371/journal.pone.0155318)

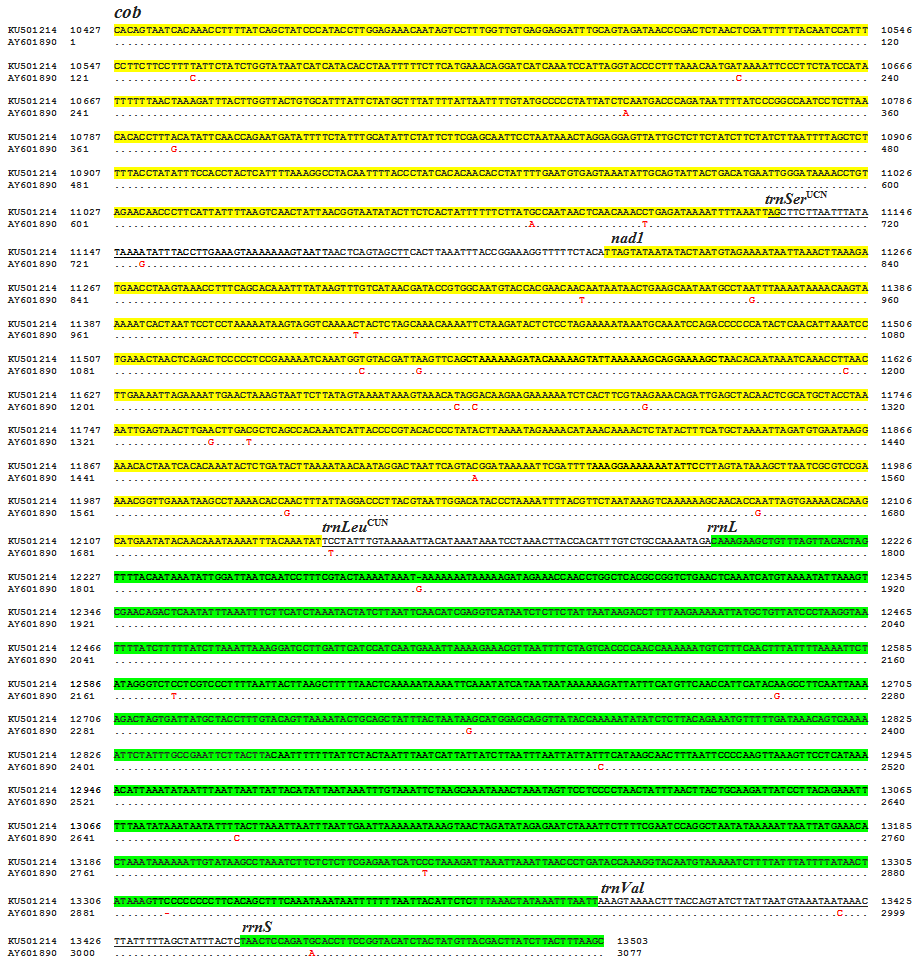

Supplement: S1 Fig — cob = cytochrome oxidase b, nad = NADH dehydrogenase subunits, rrnL = large ribosomal RNA subunit rrnS = small ribosomal RNA subunit. Sequence codes: yellow = protein coding genes, green = rRNA genes, and underlined = tRNA genes. Identical nucleotides are represented by dot “.”, and single nucleotide polimorphisms are indicated by letters in red. (TIF) [file pone.0155318.s002.tif]
